# Nonhuman Primates and Humanized Mice for Studies of HIV-1 Integrase Inhibitors: A Review

**DOI:** 10.20411/pai.v1i1.104

**Published:** 2016-05-31

**Authors:** Said A. Hassounah, Thibault Mesplède, Mark A. Wainberg

**Affiliations:** 1 McGill University AIDS Centre, Lady Davis Institute for Medical Research, Jewish General Hospital, Montréal, Québec, Canada; 2 Division of Experimental Medicine, Faculty of Medicine, McGill University, Montréal, Québec, Canada; 3 Division of Microbiology and Immunology, Faculty of Medicine, McGill University, Montréal, Québec, Canada

**Keywords:** nonhuman primates, integrase strand transfer inhibitors, integrase, drug resistance mutations, simian immunodeficiency virus (SIV), simian-human immunodeficiency virus (SHIV), simian-tropic human immunodeficiency virus (stHIV-1)

## Abstract

Since the discovery of the first inhibitors of HIV replication, drug resistance has been a major problem in HIV therapy due in part to the high mutation rate of HIV. Therefore, the development of a predictive animal model is important to identify impending resistance mutations and to possibly inform treatment decisions. Significant advances have been made possible through use of nonhuman primates infected by SIV, SHIV, and simian-tropic HIV-1 (stHIV-1), and use of humanized mouse models of HIV-1 infections. In this review, we describe some of the findings from animal models used for the preclinical testing of integrase strand transfer inhibitors. These models have led to important findings about the potential role of integrase strand transfer inhibitors in both the prevention and treatment of HIV-1 infection.

## BACKGROUND

Simian immunodeficiency virus (SIV) is naturally endemic to a wide variety of African non-human primates (NHPs). In its natural host, sooty mangabeys (*Cercocebus atys*; SM) and African green monkeys (*Chlorocebus sabaeus;* AGM), SIV infection is nonpathogenic. Notably, SIV replicates actively in infected NHPs, but unlike HIV-1 infected individuals, these animals do not generally develop immunodeficiency-like symptoms [1, 2].

Following an accidental cross-species transmission of a variant of SIV termed SIVsm to rhesus macaques, a lethal disease was observed in these hosts with symptoms similar to AIDS [3–8]; this has given rise to the pathogenic SIVmac strain [5, 9]. Cross-species transmissions from chimpanzees and SMs to humans have given rise to HIV-1 and HIV-2, respectively [10–12]. Chimpanzees have been found to develop simian AIDS (SAIDS) when naturally infected with SIVcpz, a virus that infects Central African chimpanzees (*Pan troglodytes troglodytes*) [13]. Although HIV-1 and SIVcpz share a very high degree of similarity, chimpanzees are not convenient NHP models due to their endangered status and high maintenance costs.

SAIDS and human AIDS share similar symptoms that include acute and progressive loss of CD4^+^ T cells followed by immunodeficiency, opportunistic infections, and development of tumors [14]. Routes of transmission of SIV infections in chimpanzees and macaques are similar to HIV infections in humans and include mucosal spread via vaginal [15], rectal [16], and penile routes [17] or oropharyngeal transmission in neonates [18].

Through the use of highly active antiretroviral therapy (HAART), HIV-1 infection has become manageable and is often now a chronic disease. HAART provides treatment options for both treatment-naïve and treatment-experienced patients. There are six classes of antiretroviral agents that can be used, and these are often combined in treatment. These include nucleoside reverse transcriptase inhibitors (NRTIs), non-nucleoside reverse transcriptase inhibitors (NNRTIs), protease inhibitors (PIs), integrase strand transfer inhibitors (INSTIs), fusion inhibitors (FIs), and chemokine receptor antagonists (CCR5 antagonists).

INSTIs are the most recent class of antiretroviral (ARV) drugs and include the FDA-approved agents raltegravir (RAL), elvitegravir (EVG), and dolutegravir (DTG). A new investigational INSTI, cabotegravir, which is an analog of DTG, is being developed as an oral tablet for once daily dosing and can be administered as a long-acting parenteral formulation (cabotegravir LA).

**Figure 1.**
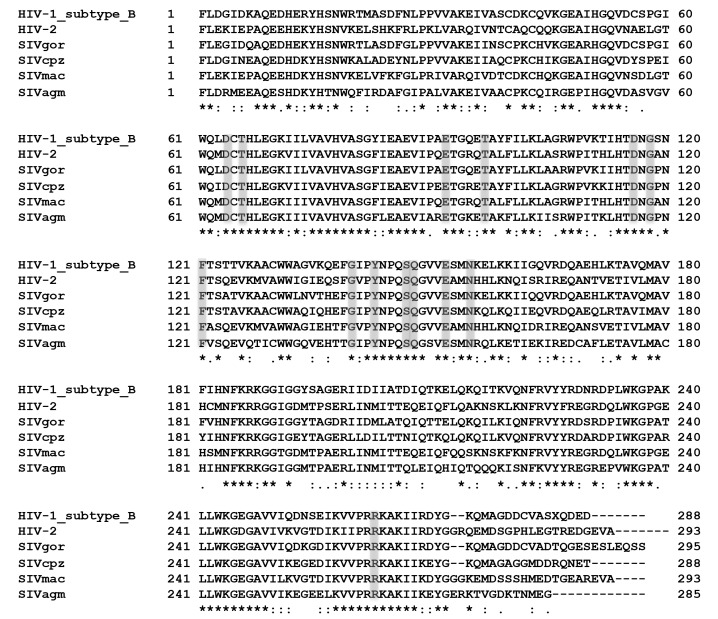
**Multiple amino acid sequence alignment of HIV-1 subtype B and different SIV integrases.** The HIV-1 subtype B integrase sequence is provided as a reference. Identical amino acid residues that are conserved across all six proteins are marked with an asterisk (*); residues similar to each other are marked with a colon (:); those that are less similar are marked with a period (.); and those that are not similar are not marked. Catalytic triad residues (D64, D116, and E152) and residues involved in main resistance pathways against integrase inhibitors (T66, E92, T97, G118, F121 G140, Y143, S147, Q148, N155, and R263) are shaded in gray. The GenBank accession numbers for the sequences used in this alignment are DQ676870 (HIV-1 subtype B), DQ307022 (HIV-2) FJ424864 (SIV_gor_CP2139.1con), EF394356.1 (SIV_cpz_Tan1.910), M33262 (SIVmac239), and U58991 (SIV_agm_Tan-1). The multiple sequence alignment was performed using Clustal Omega software [27–29].

All integrase inhibitors approved to date inhibit the integration process [19, 20]. Integration into host cell DNA is the last step performed by the HIV and SIV integrase proteins before an irreversible infection takes place in a cell. Integration occurs via two reactions that are catalyzed by the viral integrase (IN) enzyme following reverse transcription. First, IN cleaves a dinucleotide from each viral DNA terminus (long terminal repeat [LTR]) to produce reactive 3′-end processed DNA (a step referred to as 3′ processing), which is then covalently linked to the host DNA in a process known as strand transfer [21, 22]. IN contains three domains: N-terminal, catalytic core (cc), and C-terminal domains. Each domain is essential for integration. The catalytic core domain encompasses the catalytic triad—Asp_64_, Asp_116_, and Glu_152_ (D_64_D_116_E_152_) —that coordinates two divalent metal cations (Mg^2+^ or Mn^2+^). INSTIs function by disrupting the interaction between IN and viral and/or target DNA and/or chelating metal ions in the catalytic core domain [23]. Sequence alignments of the IN proteins of different SIV isolates with various groups of HIV-1 show high sequence conservation of the catalytic triad and key residues involved in resistance to INSTIs (Figure 1) [24]. Amino acids E92, T97, G118, F121, G140, Y143, S147, Q148, N155, and R263 are conserved among HIV-1, HIV-2, and different SIVs [25, 26].

In clinical trials, four primary major resistance pathways confer cross resistance to RAL and EVG, and these include substitutions at positions E92Q, Y143R, Q148R/K/H, and N155H, as well as other mutations. Q148 plus two or more additional substitutions can also decrease the clinical efficacy of DTG [30].

## ANIMAL MODELS

Several key factors must be taken into account when choosing an animal model for the study of HIV-1 pathogenesis (Table 1). These include the use of the CD4 receptor and the use of either the CC-chemokine receptor 5 (CCR5) or the CXC-chemokine receptor 4 (CXCR4) as co-receptors, nuclear export factors, transcription factors, and cellular host factors [31]. The most suitable animal models that have received broad acceptance in the HIV field include humanized mice, nonhuman primates (macaques), and to some extent cats. Although not commonly used, the feline model was valuable in the development of one of the most commonly used ARVs, tenofovir (TFV; NRTI) [32–35]. Previous research showed that feline immunodeficiency virus (FIV) DNA integration into the host cell genome can be inhibited by the INSTI naphthyridine carboxamide, L-870810, and this resulted in attenuated replication in the feline lymphoid cell line MBM [34, 36]. Animal models are important for preclinical drug testing, the demonstration of the benefits of early treatment, and the significance of drug resistance mutations.

**Table 1.** Advantages and disadvantages of surrogate models and challenge viruses for HIV-1 Infection

Surrogate Models for HIV-1 Infection	Advantages and Uses	Disadvantages
Cat	Sensitivity of FIV RT in infected cats to adefovir (N[t] RTI) helped lead to the development of Tenofovir [35, 43, 44] Susceptible to integrase inhibitors [33, 34]	Limited sensitivity to NNRTIs and PIs [45] Lacks certain genes found in HIV (*vpr*, *vpu*, Tat, *nef*) Uses CD134 as a primary receptor instead of CD4 [46] FIV can infect CD8+ T cells and B cells
Humanized mice	Direct injection using HIV-1 Informative studies demonstrating the efficacy of antiretroviral therapy [47–51]	Limitation for studying mucosal transmission of HIV-1 Cannot fully recapitulate dynamics of HIV-1 pathogenesis Cannot be bred These animals are immuno-compromised before initiation of studies
Nonhuman primates (macaques)	Infection progresses to simian AIDS Establishment of viral reservoirs Documentation of elite controllers and long-term nonprogressors Virological suppression achieved when ARVs are used Pigtail macaques mimic the menstrual cycle of hu-mans Early seeding of viral reservoirs prior to viremia [52]	Disease progression can be faster than in humans SIV and SHIV replication efficiency and pathogenesis are species dependent

Challenge Virus	Advantages and Uses	Disadvantages
SIV	Sensitive to INSTIs [25, 26, 53] Can be used for preclinical selection for integrase resistant mutants [53, 54] Utilizes CD4^+^ CCR5 T cells for replication	Limited sensitivity to NNRTIs and PIs [26, 55] Encodes for *vpx* and not the *vpu* accessory gene Lower sequence homology in some genes than for HIV Inability to replicate in human cells/cell lines Most SIVs cannot use CXCR4 Pathogenesis is species dependent
SHIV	RT-SHIVs (contain HIV-1 RT gene) are sensitive to NNRTIs Can investigate CCR5 and/or CXCR4 inhibitors and HIV-based vaccines using Env-SHIV (R5 and X4 tropic) Env-SHIV can be used to study early events following transmission	Lower sequence homology in some genes than for HIV Pathogenesis is species dependent
Simian tropic-HIV (stHIV-1)	Genome is 88% HIV-1 derived [56] Ability to infect and replicate in both human and rhesus cells by evading restriction factors [56, 57]	Inability to initiate peritoneal infection of various macaques

### BLT and SCID-hu Mice

HIV-1 cannot ordinarily infect rodents due to the inability of the HIV-1 envelope (*env*) to utilize rodent cell surface molecules for binding and entry [37] and the inability of the murine cyclin T1 protein to associate with HIV-1 Tat [38]. However, two mouse models have been developed that are now important tools for HIV research. The bone, liver, and thymus (BLT) model involves the transplantation of bone marrow, liver, and thymus tissues from humans into mice. Another model is the severe combined immunodeficiency (SCID-hu) mouse in which mice are homozygous for the SCID defect [39, 40]. This model is constructed by implanting human fetal liver and thymus under the mouse kidney capsule. Since tissue transplantation allows for HIV-1 to infect mice, many aspects of HIV-1 pathogenesis, transmission, and tissue dissemination can be addressed using these models.

Following infection with CCR5- or CXCR4-tropic HIV, successful reproduction of HIV-1 pathogenesis in humanized mice with substantial plasma viremia and systemic depletion of human CD4^+^ T cells was observed [41, 42].

### Nonhuman Primate Models

The use of animal models has helped in the preclinical evaluation and development of antiretroviral therapy (ART) and potential vaccines. Despite their genetic proximity to humans that makes them ideal as animal models for infectious diseases, the utilization of chimpanzees and gorillas poses ethical, scientific, and economic problems, with the use of the former having been mostly restricted for study of hepatitis B, C, and E viruses [58, 59]. Three macaque species have been widely used to investigate aspects of SIV infection that include viral dynamics, immune responses, and changes in CD4^+^ T cells to shed light on mechanisms of HIV-1 pathogenicity, transmission, prevention, and therapy. Although macaques are genetically more distant from humans than chimpanzees, they are widely used because they are small, easy to handle, and immunologically similar to humans. These species are rhesus (*Macaca mulatta*), cynomolgus (*Macaca fascicularis*), and pigtail (*Macaca nemestrina*) macaques [44]. These nonhuman primates are also anatomically and genetically closer to humans than cats and mice. Rhesus macaques are seasonal breeders, whereas pigtail macaques are similar to humans with lunar menstrual cycles and changes in hormone levels [60]. As a result, pigtail macaques are more suited than other macaques to capture potential fluctuations in susceptibility to SIV infection that are associated with different phases of the menstrual cycle [61–63].

All the aforementioned species are susceptible to SIV infection and develop AIDS-like disease, but rhesus macaques have been most widely used for SIV infection and have provided insights to viral transmission, pathogenesis, and latency [3, 64]. A low-dose virus challenge macaque model has several advantages that include the following features: a simian-human immunodeficiency viruses (SHIV) inoculum dose similar to physiological HIV-1 RNA levels found in semen, twice weekly virus challenges to mimic high-risk human exposure, and a SHIV_SF162p3_ isolate that utilizes an R5-tropic envelope similar to that found in most HIV-1 transmissions [65–68]. Intrarectal SIV challenge of rhesus macaques has demonstrated that the viral reservoir is rapidly seeded prior to viremia in macaques even when animals were treated with suppressive antiretroviral drugs shortly after infection [52].

## SIMIAN VIRAL AND CHIMERIC CONSTRUCTS

**Figure 2.**
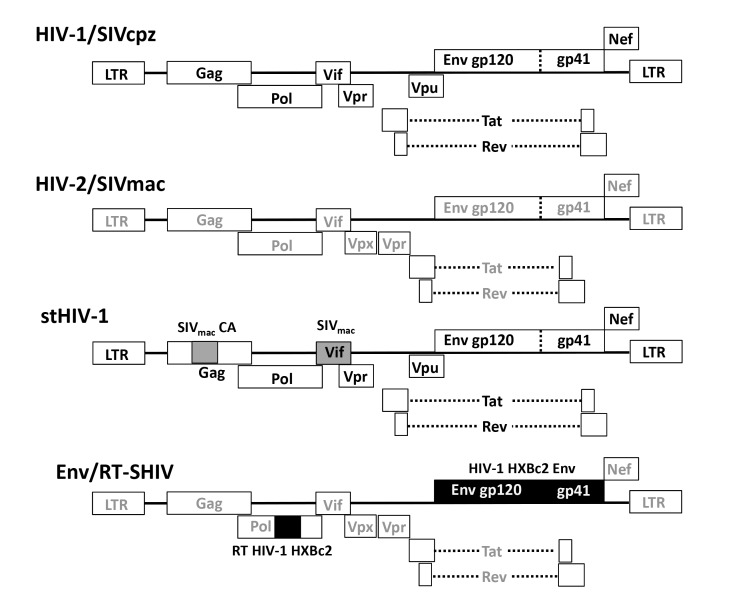
**Schematic diagram of the genomic organization of HIV-1, HIV-2, SIV, stHIV-1 and SHIV viral genomes.** HIV-1/SIVcpz encodes the *vpu* gene but lacks the *vpx* gene. HIV-2/SIVmac encodes *vpx* but not *vpu*. The *gag* polyprotein encodes matrix (MA), capsid (CA), and nucleocapsid (NC). The *pol* genomic region encodes the viral enzymes protease (PR), reverse transcriptase (RT), and integrase (IN). Gray- and black-shaded boxes indicate SIV- and HIV-derived sequences, respectively. Abbreviations: SIV, simian immunodeficiency virus; stHIV-1, simian-tropic human immunodeficiency virus; SHIV, simian-human immunodeficiency virus; SIVcpz, chimpanzee-derived; SIVmac, macaque-derived; RT, reverse transcriptase; LTR, long-terminal repeat; *env*, envelope glycoprotein.

SIV and chimeric viral constructs have been engineered with different HIV genes cloned into SIV backbones to yield SHIVs [69]. The reverse has also been done, giving rise to simian-tropic HIV (stHIV-1) [56]. The genomic organization of HIV-1, HIV-2, SIV, SHIV and stHIV-1 viral genomes is shown in Figure 2.

The HIV genome contains three major genes that encode major structural proteins and enzymes essential for replication: *gag*, *pol*, and *env*. In addition, the HIV genome contains the essential regulatory elements, *tat* and *rev*, as well as accessory regulatory proteins, *nef*, *vpr*, *vif*, and *vpu*. SIV has a similar genetic makeup as HIV except that it lacks the *vpu* gene. It encodes an additional *vpr*-related protein that is termed *vpx* that functions in association with *vpr*. Similar to HIV, SIV is unable to replicate unless integration of viral DNA takes place. Although most HIV-1 and SIV isolates use the CCR5 co-receptor to gain entry into cells, HIV-1 can acquire the ability to utilize CXCR4, whereas SIV rarely gains this ability but can utilize other co-receptors [70]. Some of the advantages and disadvantages of different viral strains and constructs are highlighted in Table 1.

The SIV/SHIV macaque model has been shown to be superior to mouse models and other non-human primates. Both HIV and SIV infections share similarities that include: 1. viral replication is suppressed by ART; 2. reservoirs of latently infected cells can persist post ART; 3. the course of viremia is characterized by an acute peak followed by a post-peak decline [71]; 4. viral transmission can occur through vaginal and rectal routes, mimicking the sexual transmission of HIV-1, or by oral routes, mimicking the transfer of virus via breast milk from mother to child; 5. the primary targets of infection are memory CD4^+^ CCR5^+^ T cells; 6. the major restriction factors that protect cells against viral replication are similar (e.g., tetherin, APOBEC3, SAMHD1, and TRIM5α); 7. there are both elite controllers and long-term nonprogressors [71]; 8. life-threatening opportunistic infections are associated with progressive failure of the immune system.

## SIV

The two most commonly used challenge SIV strains in nonhuman primates are SIVmac251 and SIVmac239, which were isolated from their natural host, the rhesus macaques. These viruses are able to cause high viral loads with minimal variations between animals. SIVmac239 was derived from SIVmac251 by animal passage and tissue culture proviral DNA cloning [9]. These viruses are widely used since they are susceptible to NRTIs, some PIs, and INSTIs, while showing relative lack of sensitivity to NNRTIs [25, 26, 55].

A three-dimensional (3D) structure solved by X-ray diffraction with a 3 Å resolution of the SIV-mac251 IN containing the solubility mutation F185H shows a contiguous core and DNA-binding domain (DBD) encompassing amino acid residues 50-293 (containing the catalytic core and C-terminal) in a single polypeptide chain (Protein Data Bank [PDB] ID 1C6V) [72, 73]. This structure shows high conservation of secondary structures (specifically in the catalytic core domain) in comparison to other retroviral Ins, including HIV-1 IN [72]; the susceptibility of SIV to different INSTIs can be attributed to the conservation of the catalytic core domain and key residues of IN.

### SHIV Expressing HIV-1 *env*

SHIVs expressing HIV-1 *env* have been widely used as challenge viruses for testing the efficacy of potential vaccines, topical microbicides, entry inhibitors, and fusion inhibitors to block viral transmission. Different SHIV viruses were designed to answer key aspects of HIV-1 replication and infection. SHIVs have also been used to assess ARVs. In recent experiments, SHIVs utilizing any of CXCR4, CCR5, or both as co-receptors have been studied. SHIV_89.6P_ is a pathogenic CXCR4 tropic SHIV clone that contains *tat*, *rev*, *vpu*, and the *env* gene of HIV-1 in a SIVmac239 background [74]; this SHIV displays a different phenotype in macaques compared to HIV-1 and SIV infections in humans and macaques, respectively [75]. The CCR5-tropic virus, SHIV_162P3_ (SIVmac239 backbone with an HIV-1 subtype B CCR5 tropic envelope [76]), is a virus transmissible in rhesus macaques by mucosal (vaginal and rectal) and parental routes of inoculation.

### Simian-tropic HIV (stHIV)

Animal models may allow for the direct study of drug resistance mutations (DRMs) and their effect on treatment success [77], but there is no model that recreates all aspects of HIV infection in humans [56]. By replacing the HIV-1 capsid (CA) and *vif* regions of the genome with the corresponding counterparts from SIVmac239 [56, 78, 79], the HIV-based chimera (stHIV-1_(SCA, SVIF)_) is capable of infecting human and macaque cell lines by escaping various restriction factors, in particular TRIM5α and APOBEC3G [56]. Few amino acid substitutions and few silent mutations in the *gag* and *pol* genes are present in the chimeric viral construct [56]. Since the active site of the integrase coding region of HIV-1 is conserved in stHIV-1, the latter has been used in studies on INSTIs and DRMs [57].

### Integrase Inhibitors

Animal models have provided important information with regard to the prophylactic efficacy of oral and topical pre-exposure prophylaxis (PrEP) with ARV drugs when animals were challenged through different routes of mucosal exposure. These models have permitted the investigation of parameters that affect ARV efficacy, such as drug resistance, drug pharmacokinetics, and pharmacodynamics.

Early studies showed that EVG suppressed the replication of SIV *in vitro* and had antiviral activity against SIV (0.5 nM [nanomolar]) [80]. *In vitro* studies also showed that SIVmac239 was susceptible to various INSTIs at nM IC_50_ concentrations, and IN mutant viruses and purified recombinant SIVmac239 IN enzymes displayed similar resistance profiles as did HIV-1 in regard to RAL, EVG, and DTG [24, 54]. This has included studies of the major RAL and EVG resistance pathways that include mutations at positions Y143, Q148R, and N155H in IN [24, 54]. DTG was shown to be more potent against IN mutant viruses and purified recombinant SIVmac239 IN enzymes than RAL and EVG [24, 54]. Tissue culture selection experiments performed using rhesus peripheral blood mononuclear cells (PBMCs) infected with SIVmac239 led to the emergence of R263K and E92Q mutations in IN for DTG and EVG, respectively [24, 54]. R263K is a non-polymorphic mutation that has also been found in several INSTI-naïve ART-experienced patients who received DTG as therapy after failing other drugs [81] and as a secondary mutation after failure with RAL and EVG [82, 83]. E92Q has been characterized as a non-polymorphic mutation that can be selected in patients receiving either EVG [84–88] or RAL [83, 84, 89] and is associated with virological failure on EVG-based regimens [89].

Previous *in vitro* studies have shown that stHIV-1 and HIV-1 also share similarities with regard to the impact of DRMs on resistance against INSTIs and on viral replicative capacity after the introduction of relevant resistance-associated substitutions into stHIV-1 [57]. The G118R and R263K IN substitutions were the most detrimental substitutions regarding stHIV-1 infectivity and replication capacity, which has also been observed for HIV-1 [90–95]. The E92Q, G118R, Y143R, N155H, and R263K substitutions in stHIV-1 conferred similar levels of resistance against INSTIs as in HIV-1 [91–99].

### Raltegravir (RAL)

The efficacy of L-870812, an INSTI, was evaluated in rhesus macaques infected with SHIV_89.6P_ and the integrase-coding genes of viruses isolated from both treated and untreated macaques have been sequenced [53]. The first report of antiviral activity of an integrase inhibitor (L870812) in SHIV-_89.6P_-infected rhesus macaques showed that this drug exhibited antiviral activity against HIV and SIV with IC_95_s of 250 and 350 nM, respectively [53]. Macaques treated early with L-870812 exhibited minimal/transient decreases in CD4 cells; four of six animals were virally suppressed to undetectable levels, while the other two treated animals did not achieve suppression but showed no decline in CD4 cell count and maintained low viral loads [53]. In contrast, animals treated later with L-870812 showed both reductions in viral load and decreases in CD4 cell count [53]. In the untreated arm, the integrase coding sequence remained unchanged, whereas N155H-harboring viruses were detected as early as 25 days in the treatment arm in the absence of noticeable viral RNA rebound or CD4 cell depletion [53]. When the N155H substitution was introduced into an HIV-1 HXB2 plasmid, N155H mutant viruses displayed both drug resistance and a reduction in infectivity [53].

Another study determined the safety of hematopoietic stem cell transplantation in ART-suppressed and unsuppressed animals [100]. In this study, they investigated the development or lack of ARV resistance after bone marrow transplantation in three groups of pigtail macaques treated with a combination of ART that includes RAL (group 1: challenged with SHIV-1157ipd3N4 [R5-tropic SHIV]) but no bone marrow transplant (control group); group 2: transplanted subsequent to SHIV challenge; group 3: challenged with SHIV post-transplant [100]. In the group 3 animals, the N155H mutation was detected within 3-9 weeks of ART initiation, and the N155H mutation was present in 71% of total IN sequences [100]. This study has some implications for scheduled treatment interruption studies in patients on ART post-bone marrow transplants, including an incomplete transplant recovery, and potential impaired viral control resulting from premature scheduled treatment interruption that may promote drug resistance [100].

Investigators have also evaluated topical prophylaxis using integrase inhibitors (L-870812 or RAL) in PrEP and post-exposure prophylaxis (PEP) in repeat low-dose vaginal challenge macaque studies [101]. To assess the window for inhibition by reverse transcriptase inhibitors and integrase inhibitors, the group performed time of drug addition experiments using HeLa-derived TZM-bl cells using a single cycle infection with vesicular stomatitis virus (VSV)-pseudotyped HIV-1 [101]. Reverse transcription occurred 1-2 hours post infection and integration more than 6 hours after infection. In this study, TFV conferred high levels of protection (> 95%) up to 2 hours after infection and ~50% protection when added 5 hours after infection, while RAL provided high protection levels (>90%) when given at 6 hours post infection and more than 50% protection when administered at 10 hours post infection [101]. The authors concluded that INSTIs may be more suitable candidates than reverse transcriptase inhibitors for prophylaxis. RAL was also examined as a topical integrase inhibitor *in vivo* after vaginal SHIV challenge. During a 10-week follow-up period, five of six macaques treated with RAL gel 3 hours post SHIV exposure remained uninfected, even after 20 weeks, whereas all four macaques that received placebo gel became infected by week 10 [90]. Sequence analysis of the integrase gene of the breakthrough RAL infection revealed wild-type genotypes despite twice weekly dosing for 8 weeks after infection [101]. The group also documented rapid vaginal absorption of RAL demonstrating a short pharmacological lag time; they also noted substantial reductions in vaginal viral load in the breakthrough infection after RAL gel treatment [101]. This study eloquently showed that protection could be achieved and that strand transfer inhibitors have a selective advantage over RT inhibitors since they provide an optimal window for post-coital dosing, which was not a viable option with entry or RT inhibitors [102, 103].

Treatment of SIVmac251 in cultured MT-4 and CEMx174 cell lines by RAL showed inhibition with IC_50s_ in the low nM range [25]. RAL-monotherapy (50 or 100 mg RAL twice daily with food) of SIVmac251-infected rhesus macaques for 10 days resulted in a decrease in viral load [25], while the addition of emtricitabine (FTC) and TFV to the treatment led to undetectable viral loads and CD4 cell increases within 2 weeks [25]. However, proviral DNA levels did not change and persisted in PBMCs during the treatment period, indicating the persistence of viral reservoirs [25].

## MOUSE STUDIES

In an early study with humanized mice, L-870812 displayed similar outcomes as in humans, causing suppression of viremia below the limits of viral RNA detection and recovery of CD4^+^ T cells [104]. In this same study, an interruption of ART resulted in viral rebound and loss of CD4 T cells [104]. The authors also reported that treatment failure was associated with the appearance of drug resistance mutations with one of six INSTI-treated mice acquiring mutations associated with RAL resistance at positions D55N, E92Q, E152I, and M154I within IN [104]. As mentioned above, E92Q is a mutation that is associated with resistance to RAL in humans [83, 84, 89]. Substitutions at position E152 in IN are significant since this residue is part of the catalytic triad that facilitates binding of Mg^2+^ to the active site; if the IN viral protein is inactive as a result of mutations at this position, then the integration step will not take place and viral replication may be arrested.

RAL was also examined to determine its potential as a candidate for PrEP [49]. Whereas all of the untreated control infected mice became virus positive within 5 weeks following vaginal challenge with HIV-1 BaL-1, oral administration of RAL fully protected humanized mice [49]. No evidence of infection in treated mice was detected during a 10-week period of evaluation [49]. Moreover, no viral RNA in plasma or proviral DNA in cellular fractions was detected using PCR [49].

Recently, the efficacy of long-acting (LA) RAL to protect against vaginal HIV transmission in a PrEP study was investigated. Using transmitted/founder HIV, researchers observed that a single subcutaneous administration of LA RAL to BLT mice provided protection against two high dose HIV challenges at 1 and 4 weeks after drug administration [105]. Researchers also demonstrated penetration of RAL into the female reproductive tract. In addition, these studies documented viral RNA suppression in plasma and in the cervico-vaginal fluids of BLT-infected mice [105]. In mice infected with HIV-1_CH040_ and HIV-1_RHPA_ transmitted/founder (T/F) viruses did not possess detectable mutations were detected in viral DNA derived from plasma. In the case of one infected mouse infected by a HIV-1_THRO_ T/F virus, a single amino acid substitution, I268L, was identified in the integrase gene [105]; although the aforementioned mutation has not previously been associated with RAL resistance [106], one patient receiving RAL treatment did develop a I268M substitution in addition to T97A, Y143R, and other substitutions [107].

Similar trends were seen when studies on mucosal tissue pharmacokinetics of RAL in humanized mice were carried out as in human studies; RAL exhibited higher drug exposure in vaginal and rectal tissues relative to plasma and higher exposure in intestinal mucosa than in plasma [51].

### Elvitegravir

Elvitegravir (EVG) alone is not widely used in animal studies since it was originally approved as part of the fixed dose combination known as Stribild (combination of elvitegravir/cobicistat/emtricitabine/tenofovir disoproxil fumarate), and researchers were mostly interested in identifying drug resistance, drug pharmacokinetics, and pharmacodynamics of single formulation drugs [88]. However, EVG was recently approved by the FDA as a single pill formulation for ARV-experienced patients [108]. While EVG alone may not have been used in PrEP or monotherapy studies using animal models, pharmacokinetic studies showed that the drug attained higher penetration levels in rectal and vaginal fluids in rhesus macaques despite the absence of pharmacological boosting than were achieved with RAL or DTG [109]. Rectal secretions collected from EVG-treated macaques showed higher antiviral activity than did those from DTG- or RAL-treated mice in TZM-bl cell assays [109].

### Cabotegravir

Cabotegravir (CTG), a DTG analog, is another INSTI currently under development that possesses favorable pharmacokinetics, safety, and efficacy profiles in the clinic [110–112]. While DTG possesses conformational flexibility (6-membered ring) of the metal-chelating scaffold, CTG has a more rigid scaffold (5-membered ring). The half-life of a long-acting (LA) form of CTG was shorter in macaques (3 to 12 days) than in humans (21-50 days) [110, 113]. CTG exhibited high potency against HIV-1 BAL strains in human PBMCs [113]. Due to its high potency, slow metabolism, and highly protein bound, CTG lends itself to use as a LA injectable suitable for monthly to quarterly clinical administration [110, 113, 114]. CTG formulation is also being developed as a single agent for PrEP. In animal studies, the LA form of CTG has been shown to protect macaques from repeated low dose intrarectal SHIV challenges, thereby demonstrating proof of concept of LA CTG in PrEP [115]. Eight macaques were intramuscularly injected with 50 mg/kg of CTG LA at two time points (before and after challenge) before intrarectal SHIV_162p3_ repeated challenge [115]; whereas treated macaques remained aviremic during the challenge and wash-out periods, untreated macaques became infected following challenge [115].

Another experiment was performed to determine the minimal drug level needed to achieve protection following low dose intrarectal SHIV challenge. Treated macaques were injected once per week before SHIV challenge, while control macaques did not receive any treatment [115]. Control macaques became infected after 1-2 virus challenges, while treated animals were infected only after 6-17 virus challenges, which coincided with a decline in plasma drug concentrations [115]. Cell-free plasma samples from CTG-treated macaques were also analyzed for integrase amino acid substitutions. Although no primary integrase resistance mutations were observed, substitutions at positions G27R, A122T, E173K, and D256E were detected [115]. It has been postulated that once monthly intramuscular injection of CTG (50 mg/kg) might be able to reach the same high plasma drug concentrations that are achieved in humans following an 800-mg intramuscular injection [115].

In another experiment, it was shown that a single dose of LA CTG delayed infection in repeated high-dose intravaginally SHIV-challenged macaques [116]. Animals receiving placebo became infected at 1 to 2 weeks post SHIV_162P3_ challenge, whereas protection was observed for 6 of 8 CTG-LA-treated rhesus macaques against three high-dose SHIV challenges (on weeks 1, 5, and 7 following LA CTG intramuscular injection) [116]. Consensus sequence analysis of the SHIV integrase-coding regions from the plasma of infected treated macaques identified P142S and I210V on different genomes at weeks 11 and E198G at week 20. These mutations did not decrease susceptibility to CTG *in vitro* [116]. Of the three mutations identified, E198 and P142 are both conserved in HIV-1 integrase. Substitutions at positions 198 and 142 have been previously reported in patients receiving RAL [89, 117, 118].

In agreement with the aforementioned study, another group demonstrated that monthly injections of CTG provided complete protection against repeated intravaginal SHIV_SF162p3_ challenges in pigtail macaques [119]. In this study, female pigtail macaques were intravaginally challenged with SHIV_162P3_ twice per week for up to 11 weeks [119]. All of the placebo controls became infected while all macaques that received CTG LA intramuscularly were protected from infection and remained seronegative as well as seronegative for viral RNA and DNA for more than 22 virus challenges [119]. Both of these studies support the clinical development of CTG LA as a PrEP candidate to prevent HIV infection.

## CONCLUSIONS

Animal models have been essential for answering key questions pertaining to antiretroviral therapy, including studies on treatment interruption, tissue biopsies to study cells and tissues, change of regimens, drug resistance-associated mutations, and investigation of novel classes of antiretroviral drugs. Because new anti-HIV drugs still need to be developed to combat drug resistance, the use of non-human primates, humanized mice, and other animal models of viral infection will continue to be essential for this endeavor, as well as for studies on viral pathogenesis.
